# Low Volume Vertebral Augmentation with Cortoss® Cement for Treatment of High Degree Vertebral Compression Fractures and Vertebra Plana

**DOI:** 10.7759/cureus.1058

**Published:** 2017-02-26

**Authors:** Robert E Jacobson, Michelle Granville, Jesse Hatgis, Aldo Berti

**Affiliations:** 1 Miami Neurosurgical Center, University of Miami Hospital; 2 Larkin Hospital, Nova Southeastern University School of Osteopathic Medicine

**Keywords:** vertebral compression fracture, vertebra plana, vertebroplasty, vertebral augmentation

## Abstract

This is a retrospective analysis of a consecutive series of patients undergoing vertebroplasty and vertebral augmentation in an outpatient setting for high degree osteoporotic vertebral fractures or vertebra plana using consistently low volumes (less than 3 cc) of Cortoss® cement, rather than polymethylmethacrylate (PMMA). The results in these patients demonstrate that it is both technically feasible to do vertebroplasty on these patients and using a low volume hydrophilic silica-based cement is effective in providing diffuse vertebral body fill with minimal complications. There was no increased risk of complications, such as cement leakage, displacement of bone fragments, or progression of the angulation. Specifically, with over a 24-month follow-up, the preoperative collapse or angulation did not worsen and none of the patients developed adjacent level fractures or required further surgery at the involved vertebral level.

## Introduction

**Figure 1:**
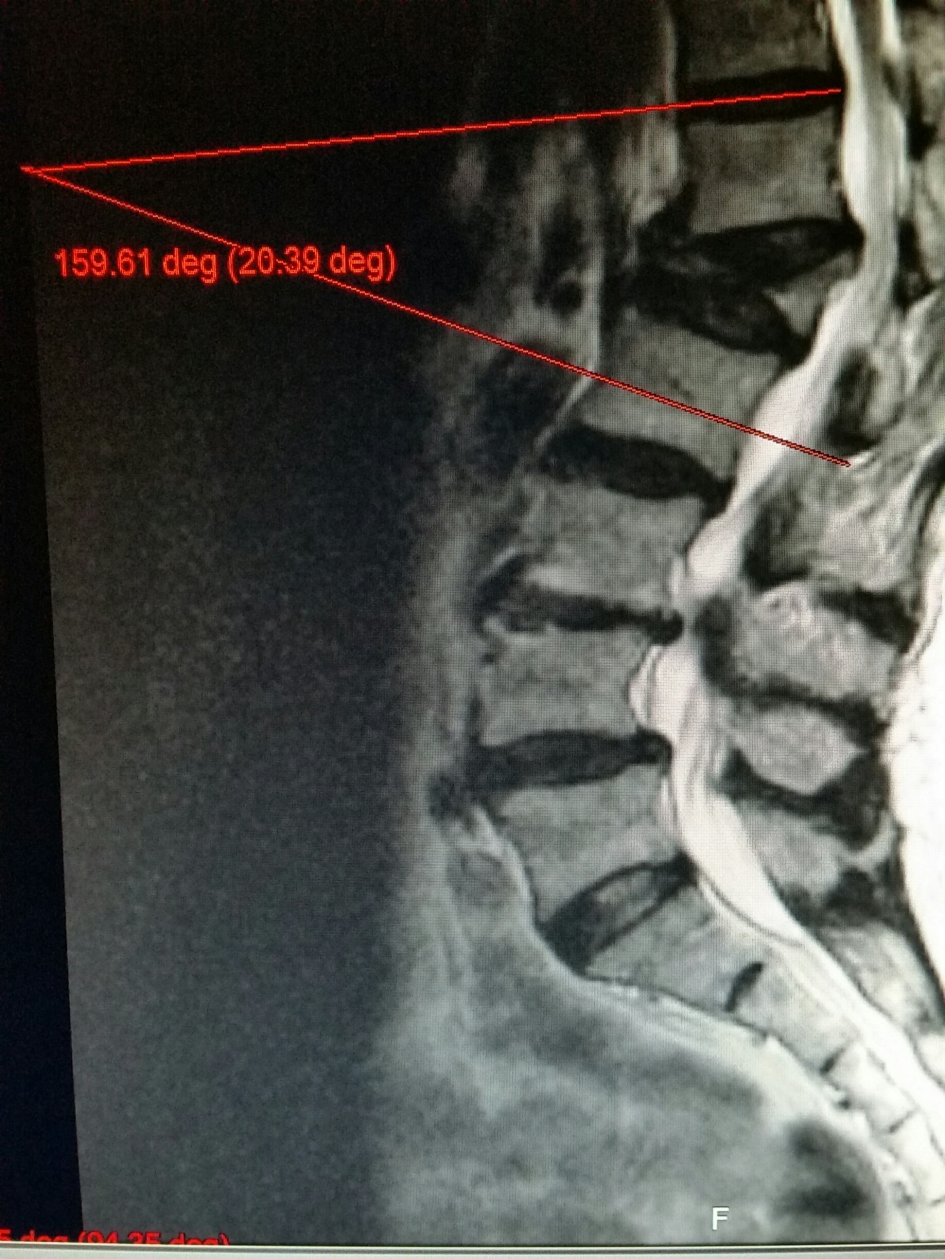
MRI sagittal view demonstrating kyphotic angulation after progressive collapse of an untreated vertebral compression fracture

High degree vertebral collapse with osteoporotic fractures has been defined as a greater than 70% loss of anterior vertebral height compared to posterior vertebral height. Vertebra plana is defined as a complete collapse of the anterior vertebral body with a minimal collapse of the posterior wall, creating a ‘pancake’ like flattening of the vertebral body, as shown in Figure 1 [1].

There is recent literature suggesting vertebral augmentation or balloon kyphoplasty are effective in high degree collapse; however, note that there are higher risks of both intraspinal and intradiscal leakage of cement [2]. All of these reports used polymethylmethacrylate (PMMA) cement, which is an inert hydrophobic polymer, with or without balloon kyphoplasty, for use in partially (30-50%) collapsed vertebral bodies [3]. In this study, we used Cortoss® (Stryker®, Malvern, PA). Cortoss® is a bioactive calcium phosphate micro-glass cement that is more bone binding and hydrophilic with a lower curing temperature than traditional PMMA [4]. Biomechanically, Cortoss® is stronger than PMMA and similar in strength to cortical bone. This allows patients to begin weight-bearing activities immediately after vertebral augmentation. 

Multilevel screw fixation and stabilization is often recommended to stabilize or correct the collapse and deformity without cement augmentation at the fracture site. There are limited reports of doing short segment stabilization combined with kyphoplasty at the collapsed spinal level and even using a calcium phosphate-based cement similar to Cortoss® [5]. However, extensive surgery, especially with screw fixation, can be problematic in these elderly patients since many have diffuse and severe osteoporosis with poor bone density making it difficult for pedicle screws to hold and maintain any kyphotic correction without adding cement to stabilize the screws. Many of these same patients are not only elderly but have multiple associated medical conditions that make extensive surgery high-risk, leading to other unintended complications.

During the widespread use of balloon kyphoplasty in the 1990’s, the assumption evolved that treatment needed to be bilateral and required larger volumes of cement, alongside the usage of expanded balloons, to attempt vertebral height restoration and vertebral angulation correction for pain relief [1, 6]. Contrary to this belief, height may only be restored in a limited percentage of acute or subacute fractures. The post-procedure restoration of height and kyphotic correction was limited and often not maintained with weight-bearing. There was also an association with a significant incidence of cement leakage and balloon breakage during the time of the procedure [1, 6]. Additionally, there does not exist a clear reported correlation between vertebral height correction and pain relief. Several authors have raised the concern that rigidity from large cement volumes leads to a higher rate of adjacent level fracture [1, 6]. 

This report will review the following: 1) technical steps of introducing vertebroplasty cannulas and cement in high degree vertebral collapse, 2) the effect of Cortoss® on these fractures, and 3) the clinical and pain responses from treated patients. 

## Materials and methods

In our practice, 76 patients underwent vertebroplasty or kyphoplasty within the last 24 months. Eighteen of these patients had greater than 70% vertebral collapse or vertebra plana. The average patient age of the entire group was 76 years, ranging from 23 years to 90 years. Female to male ratio was 4:1. The vertebral fracture levels most frequently affected were T12 and L1 in the vertebra plana group, followed by L4 and L5 in both early stage fractures and high degree fractures. Patients with a previously instrumented spine were more likely to fracture below the instrumentation if there was a previous history of untreated osteoporosis. The interval from the onset of pain to initial office evaluation was typically between two weeks and four months, with the earliest case presenting 10 days status-post a fall and the longest case presenting over three years status-post a traumatic event. Average pre-procedural visual analog scale (VAS) pain level was 8/10. Post-procedural VAS pain level was 4/10. The most common comorbidities included osteoporosis (mainly in women), prostate cancer or trauma (in men), scoliosis, kyphosis, history of bone cancer, colon cancer, and/or exposure to radiation/chemotherapy, diabetes, and hypertension. Informed patient consent was obtained at the time of treatment.

### Radiologic studies

Radiologic inclusion criteria for the review group was vertebral body collapse of greater than two-thirds height or complete vertebra plana in one or more vertebral bodies. Pre-procedural studies included DEXA scan, bone scan, MRI, and/or CT with reconstruction. Immediate postoperative AP/lateral films and follow-up CT or MRI scans were also obtained. Kyphotic angles were measured on MRI or CT scans both pre and post-procedurally. 

### Procedural technique  

All procedures were performed in an ambulatory surgical center with local anesthesia and monitored anesthesia care (MAC) sedation. Patients were positioned prone, the involved spinal level was confirmed with fluoroscopy, and the skin was marked at the level of the pedicle with a surgical pen. The skin was anesthetized with a 25-gauge needle over the identified pedicle bilaterally in an AP trajectory. Then, 20-gauge spinal needles were positioned to contact each pedicle directly, and the trajectory path was confirmed with lateral fluoroscopy. The primary side for the initial entry point was decided based on the area of maximum collapse and the ability to visualize/access the pedicle. 

**Figure 2:**
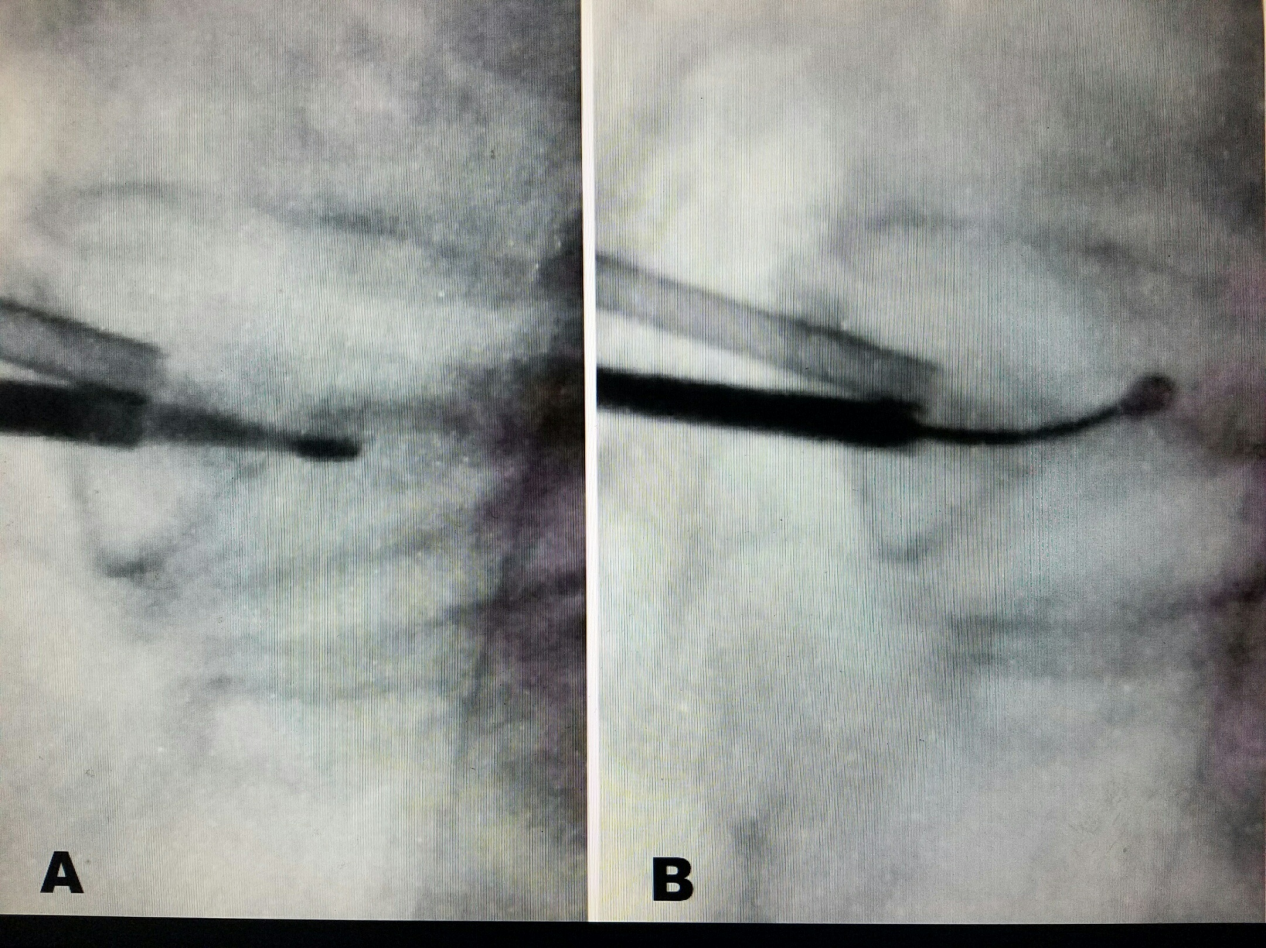
Intraoperative fluoroscopic sagittal views during vertebroplasty demonstrating access cannulas and curettes A: Instrumentation within the posterior vertebral body and pedicle. The straight curette is passed only to the middle of the vertebral body in the sagittal view. B: The curette is curved superiorly to dissect a path for Cortoss® spread during vertebroplasty.

A 2-3 mm horizontal skin incision was made adjacent to the 20-gauge needles, and an 8, 10, or 11-gauge Stryker® vertebral augmentation access cannula was inserted into the pedicle. This was performed by using either a mallet or manual hand pressure. Often, the bone was so osteoporotic that the cannula entered through the pedicle without requiring a mallet. Entry into the posterior border of the collapsed vertebral body or vertebra plana was confirmed on lateral fluoroscopy. No attempt was initially made to pass the cannula more anteriorly into the collapsed vertebra, especially in the presence of a severe vertebra plana. Next, a fine Stryker® drill was carefully inserted between 5 and 10 mm into the vertebra. The drill tip diameter measured 2 mm, which was small enough to gain access into the vertebra plana. Specimens from the drill tip were obtained for biopsy. Continuous lateral fluoroscopy was used for instrumentation access into the vertebral body midpoint, as shown in Figure 2. An expandable curved augmentation curette was then placed into the midpoint of the fractured vertebrae. It was slowly advanced medially and then positioned slightly superiorly and inferiorly for the purpose of creating a small cavity. The cannula was positioned as centrally as possible within the vertebral body.

Then, 0.3 - 0.4 cc of Cortoss® was placed through the cannula into the vertebral body via a pipette under continuous AP fluoroscopic guidance. This served as a "test" injection to determine the cement flow pattern, which most importantly allowed visualization of possible contralateral spread through the midline. If contralateral spread did not occur, then the contralateral side of the vertebral body was accessed as previously described. Thereafter, a minimum of one Stryker® pipette of Cortoss®, or 0.73 cc, was placed on each side for adequate vertebral body fill, totaling approximately 1.4 cc of Cortoss®. If midline spread occurred, then only a unilateral approach was required. Again, a minimum of 1.4 cc of Cortoss® was placed. Usually, the total amount of Cortoss® placed was between approximately 2.1 cc and 2.8 cc. All Cortoss® injections were performed under continuous fluoroscopic guidance. If any sign of posterior extravasation or venous flow was noted, the injection was immediately terminated.

Once the vertebral body was adequately filled, the cannulas were removed, the small skin incisions were closed with skin adhesive and Steri-Strips™ (3M, St. Paul, MN), and the patient was returned to the recovery room. Total operative time on average was 15 minutes for one level and 25 minutes for two or more levels. Average blood loss was less than 2 cc. Ice was routinely placed over the surgical site for approximately 30 minutes. Final hard copy films of saved intraoperative fluoroscopic images were obtained. Under proper supervision, all patients ambulated without any brace prior to discharge home from the ambulatory surgical center. Patients were typically seen for follow-up in the office between three to five days after the procedure, then six weeks and three months after the procedure. Follow up x-rays were routinely obtained prior to the six-week follow-up.

## Results

Eighteen of the 76 vertebroplasty/kyphoplasty cases performed had greater than 70% vertebral body collapse (vertebra plana) and was the basis for this review. In the 18 patients with a high degree fracture or vertebra plana, vertebroplasty and augmentation were done unilaterally in 10 and bilaterally in eight. Multiple level vertebroplasty and augmentation were approached in three of the 18 patients. In all three cases, one level had a vertebra plana or high degree collapse and the additional fracture was less than 50% at an adjacent level. Pre-procedural angulation measurements showed 10/18 patients had less than 5 degrees of kyphotic angulation, three had between 5 to 10 degrees, and five had between 10 to 20 degrees of kyphosis secondary to the vertebral compression fracture. In the three-month and six-month postoperative follow-up visits, none of the patients had increased angulation on follow-up films post-procedure.

Eighty-five percent of patients had symptomatic pain relief within 24 to 96 hours. The remainder of the patients showed improvement over seven to 10 days. Preoperative VAS scores were 7-8/10 and post-procedure averaged 4/10. At the one month follow-up, it was 1-2/10 in 12 of 18 patients. Follow-up evaluation up to two years later showed patients remained pain-free or with low VAS scores. Patients did not develop increased kyphotic angulation if present pre-procedure. There were no cases of adjacent level fracture in all 18 single-level cases followed from six to 24 months. All three patients with multilevel fractures had poorer starting bone density, and one subsequently developed a new fracture at another level but not adjacent to the high degree fracture that underwent vertebroplasty and vertebral augmentation.

The amount of cement used for vertebral augmentation was purposely limited to the lowest volume of cement showing radiologic "fill" of the collapsed vertebrae. The average volume of Cortoss® used was 1.4 cc unilaterally and a total of 2.1 cc bilaterally. There was leakage noted at the time of injection in only one case; however, two patients were found to have cement leakage into the spinal canal on routine follow-up CT scan. One patient had moderate symptoms of radiating pain to the flank on the side of leakage (less than 2 mm posterolaterally), which resolved with seven days of oral prednisone. There were no post-injection neurologic complications or infections in this group.

## Discussion

This study looked at three interconnected issues: 1) Was it technically feasible to perform vertebral augmentation in patients with such a high degree of vertebral body collapse without intra-procedural complications? 2) Would low volumes of Cortoss® be clinically effective in relieving pain and preventing progressive vertebral collapse or kyphotic deformity? 3) In follow-up evaluation, would there be a similar or reduced rate of complications, such as leakage, adjacent level fracture, or progressive kyphotic deformity, compared to kyphoplasty with higher volumes of cement or instrumentation?

There is often some degree of kyphotic angulation as the collapse progresses. It is most commonly seen in those with osteoporosis but can also be seen in a younger population associated with Paget's disease and other tumors [7]. Using detailed MRI and CT scans with reconstruction, it is possible to visualize several possible scenarios when considering performing a vertebral augmentation procedure: breach of the posterior canal wall on both sagittal and axial reconstruction, any degree of posterior fragment displacement of bone into the spinal canal, and degree of angulation. CT reconstruction demonstrates that even with a 90% collapsed vertebral body, as in vertebra plana, the collapse occurs anterior to the pedicles, which rarely collapse, although a pedicle could contain a fracture line [1].  

Balloon kyphoplasty has been recommended to create some degree of expansion of the vertebral body and attempts to correct the kyphosis in moderate (less than 50%) vertebral collapse; however, most series report only 3-10% of improvement in the kyphotic angle and height [6]. Patients with high degree vertebral body collapse may benefit from the properties of Cortoss® over traditional PMMA. Cortoss® has a lower viscosity, which allows for more diffuse spreading into the microfractures [6, 8]. Also, Cortoss® biologically forms a bond with bone and contains osteoconductive properties, thereby providing better biomechanical structural support, strength, and bone quality, all while promoting actual redevelopment of bone at the fracture sites. When combined with aggressive medical management of the patient’s osteoporosis, vertebral augmentation with the use of Cortoss® may demonstrate better long-term outcomes than traditional therapies [6].

### Accessing the collapsed vertebra

In osteoporosis, there is a progressive loss of trabeculae of cancellous bone that is replaced with weaker, less developed, and less calcified bone. There is also concurrent thinning of the cortical rim, leading to weakening of the involved vertebral body. Anatomic studies show that the vertebral body is over 95% cancellous bone, while the pedicle is consistently only 65 to 75% cancellous bone, leading to a differential weakening of the vertebral body compared to the pedicle [9]. The pedicle also has relatively more cortex than the vertebral body, and the pedicle cortical rim is thicker medially and superiorly [9]. This relatively thicker cortical rim covers a large percentage of the oval-shaped pedicle area. Biomechanical and anatomical studies demonstrate the oval shape of the pedicle, which is 11 to 15 mm longer in the sagittal vertical plane than the coronal plane, to contribute to the higher resistance to vertical compression [9]. 

**Figure 3:**
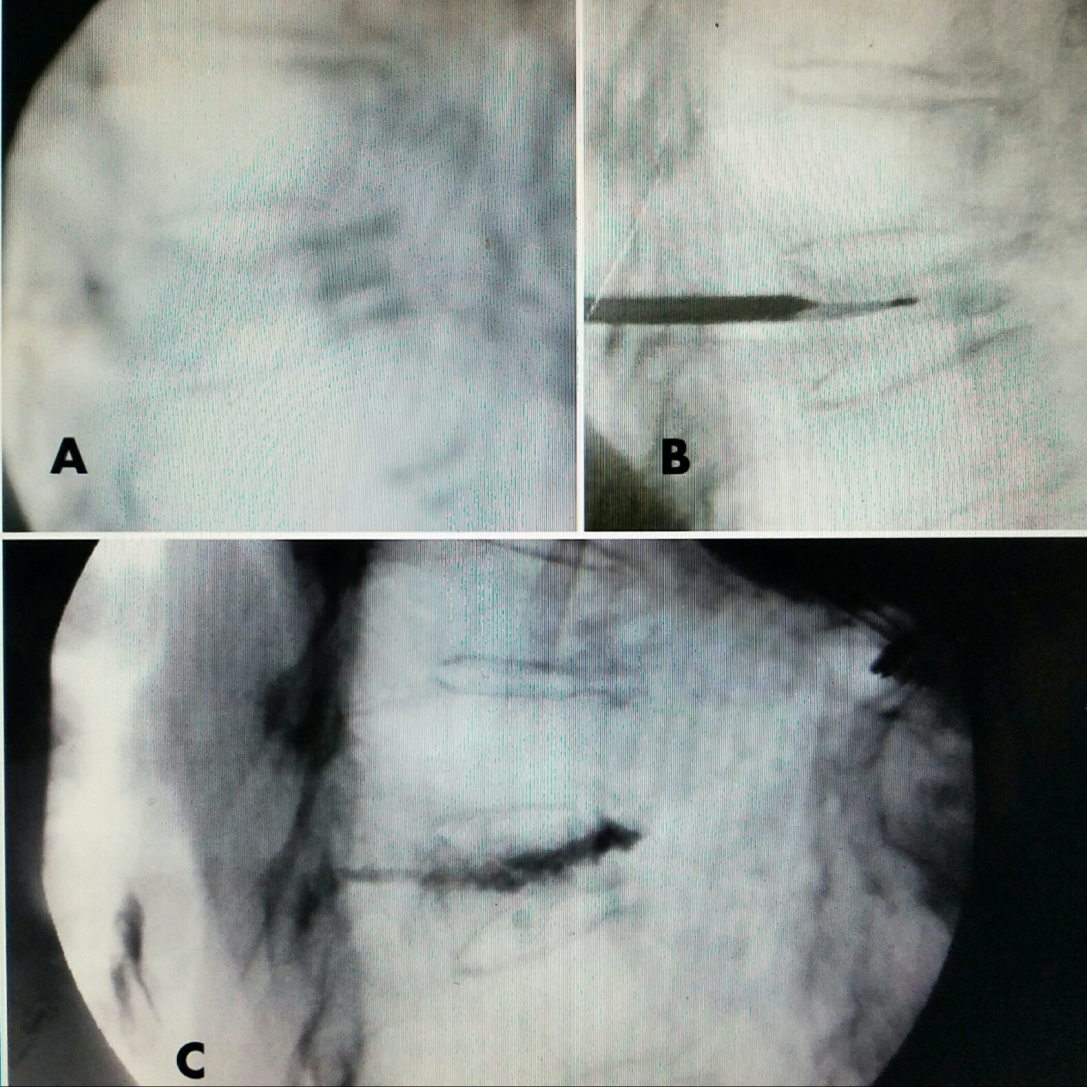
Pre and post-injection films A: T8 vertebra plana. B: Single cannula and dissecting curette slightly superior creating cavity. C: Post-injection of Cortoss® cement shows complete fill, especially anteriorly, providing support

In turn, the thicker pedicle cortex, especially medially and superiorly, provides vertical and medial support to the posterior edge and wall of the osteoporotic vertebra where the pedicles are part of the middle column of the vertebra [10]. The vertebral body forming the anterior column is almost all cancellous bone and is in the center of the normal vertical load of the spinal column. In osteoporosis, the cancellous bone becomes decalcified, softer, and weaker, resulting in more widely spaced trabeculae that are replaced with an immature matrix that is significantly less resistant to weight-bearing forces. The preservation of the stronger bilateral pedicles provides more posterior support to the posterior wall of the osteoporotically weakened vertebrae. Since the pedicle makes up approximately 30 to 50% of the posterior vertebral wall vertical height, this posterior support, combined with the cancellous anterior vertebral body, explains the classic progressive anterior wedge collapse of the osteoporotic vertebra [9]. In its final stage, the vertebrae take on a vertebra plana "pancake" shape but still retains relative preservation of the pedicles [10]. This extreme collapse of the anterior vertebral body, which has no lateral support, tends to force the collapse more towards the center. The pedicles remain normal in size, ‘partially’ supporting the posterior and posterolateral part of the collapsing vertebrae. This preservation of the pedicle height directly to the posterior part of the vertebral body is what allows access to the vertebra via a transpedicular approach in every case from at least one side [9]. This is critical for surgical access since the relatively normal-sized pedicle (11 to 15 mm vertically) allows the surgeon to “thread” the pedicle using a size 8 to 11-gauge (3.2 to 2.2 mm) access cannula to enter the collapsed vertebra [9]. After passing through the relatively intact pedicle and with the careful use of a controlled curved dissector curette (Stryker®) under constant fluoroscopic monitoring, it is possible to create a cavity and path that can be manually dissected toward the center from one or both sides of the vertebra. All dissection with the curette is directed anteriorly and medially to avoid the posterior wall. This prevents the formation of any new posterior fractures that could lead to cement leakage. The path developed with the dissector forms a small cavity within the collapsed vertebra that allows the easy flow of cement diffusely and often bilaterally for proper stabilization, as shown in Figure 3.

### Use of low volume Cortoss® compared to PMMA 

Biomechanically, another potential advantage of low volume vertebroplasty in contrast to balloon kyphoplasty is that there is minimum pressure on the adjacent vertebrae. Using a balloon bilaterally creates a larger relatively circular defect from 1.0 - 1.5 cm in size within the already softened and fractured osteoporotic vertebra. The addition of hard PMMA cement in large, inert masses potentially changes the mechanics of the collapsed vertebra by filling multiple microfractures with a larger and more rigid mass of PMMA within the balloon [2-6]. These masses potentially create pressure that leads to further endplate fractures. There is a significant percentage of balloon cases where the balloon ruptures, sending higher volumes and higher pressure cement into the vertebra [2-6]. In vertebra plana and fractures with high degree collapse, pressure from the large mass of PMMA on one or both sides can fracture the cortical borders of the collapsed vertebra or, more importantly, the endplate, thereby putting pressure on the adjacent vertebrae [2-6]. This creates stronger forces on already weakened bone at the involved vertebra as well as at the adjacent vertebrae. The balloon technique using PMMA does not allow for the cement to fill many microfractures throughout the collapsed vertebra, which does not seem to occur with vertebroplasties using Cortoss® [8].  

### Comparison of complications

Conceptually, low volume cement fixation allows the cement to flow to the various fractured areas and ‘fix’ the collapsed vertebra in place. Cortoss® studies demonstrate easier flow characteristics before the cement sets and biomechanical strength similar to normal bone [8]. By providing more diffuse, evenly distributed support without creating a local rigid mass of cement, there is less localized and more evenly distributed pressure on the adjacent vertebrae. There is less of a chance of adjacent collapse, as seen in this group of patients, although it is a small group followed up to 24 months. Since Cortoss® has been shown to possess bioactive qualities, patients could theoretically have a stronger vertebra over time, even with a collapse. This may be especially true if patients additionally have the appropriate medical management of their osteoporosis with bone-building agents. The original Cortoss® studies in cases of mild to moderate collapse (50% or less osteoporotic compression fractures) support this hypothesis, as our cases had both diffuse vertebral body fill and a lower incidence of adjacent level fractures [8].  

Our patients only had a small volume of cement injected either unilaterally or bilaterally (i.e., 1.4 cc unilaterally and 2.1 - 2.8 cc bilaterally per vertebra). Cement was used to stabilize the fracture without any attempt to “reduce the compression” with a balloon. Initial patient clinical response and follow-up demonstrate that low volume is technically feasible and effective for pain relief. It also has a lower incidence of adjacent level fractures (similar to use in fractures with lesser height loss) and does not lead to further deformity or kyphosis. The procedure can be performed in an outpatient setting with short operative times of 10 - 15 minutes for a single vertebra, which is preferable in elderly osteoporotic patients with other medical comorbidities. 

## Conclusions

This retrospective study demonstrates that satisfactory results are obtained using very low doses of Cortoss® in high degree osteoporotic vertebral collapse and vertebra plana specifically due to osteoporosis. A small amount of Cortoss® cement (averaging 2 cc), which is able to spread throughout the collapsed vertebra, is effective in relieving pain from the fracture and avoiding further kyphotic deformity. This was demonstrated in both short-term and long-term follow-up. The low dose cement effectively stabilized the fracture in situ with no documented progression of the kyphotic deformity. Additionally, since many of these patients are older and have multiple medical comorbidities, minimal anesthesia and short operative times are beneficial in avoiding complications. All cases were performed under local anesthesia with MAC sedation as needed in an ambulatory surgical center. No patient required hospitalization due to the procedure. Patients with high degree fracture and vertebra plana were specifically excluded from the original Cortoss® study, which makes the results of our study that much more significant. Our results are comparable to other procedures without the increased risk of complications from cement leakage, progression, or development of kyphotic deformity at the site of the fracture or an increased frequency of adjacent level fractures.
